# Evaluation of a public COVID-19 dashboard in the Western Cape, South Africa: a tool for communication, trust, and transparency

**DOI:** 10.1186/s12889-022-14657-w

**Published:** 2022-12-29

**Authors:** Muzzammil Ismail, Erna Morden, Hannah Hussey, Masudah Paleker, Theuns Jacobs, Inneke Laenen, Mehreen Hunter, Melvin Moodley, Mariette Smith, Themba Mutemaringa, Jamy-Lee Bam, Pierre Dane, Alexa Heekes, Andrew Boulle, Mary-Ann Davies

**Affiliations:** 1 Health Intelligence Directorate, Western Cape Government: Department of Health, Cape Town, South Africa; 2 School of Public Health and Family Medicine, Faculty of Health Sciences, University of Cape Town, 7925 Cape Town, South Africa; 3 Division of Health Systems and Public Health, Stellenbosch University, Stellenbosch, South Africa; 4 Computational Biology Division, Integrative Biomedical Sciences Department, University of Cape Town, Cape Town, South Africa; 5 Centre for Infectious Disease Epidemiology and Research, Cape Town, South Africa

**Keywords:** COVID-19, Dashboard, Western Cape, South Africa, Digital, Pandemic, Data, Evaluation, Impact

## Abstract

**Background:**

Public health dashboards have been used in the past to communicate and guide local responses to outbreaks, epidemics, and a host of various health conditions. During the first year of the COVID-19 pandemic, dashboards proliferated but the availability and quality differed across the world. This study aimed to evaluate the quality, access, and end-user experience of one such dashboard in the Western Cape province, South Africa.

**Methods:**

We analysed retrospective aggregate data on viewership over time for the first year since launch of the dashboard (30 April 2020 – 29 April 2021) and conducted a cross-sectional survey targeting adult users of the dashboard at one year post the initial launch. The self-administered, anonymous questionnaire with a total of 13 questions was made available via an online digital survey tool for a 2-week period (6 May 2021 – 21 May 2021).

**Results:**

After significant communication by senior provincial political leaders, adequate media coverage and two waves of COVID-19 the Western Cape public COVID-19 dashboard attracted a total of 2,248,456 views during its first year. The majority of these views came from Africa/South Africa with higher median daily views during COVID-19 wave periods. A total of 794 participants responded to the survey questionnaire. Reported devices used to access the dashboard differed statistically between occupational status groups with students tending toward using mobile devices whilst employed and retired participants tending toward using desktop computers/laptops. Frequency of use increases with increasing age with 65.1% of those > 70 years old viewing it daily. Overall, 76.4% of respondents reported that the dashboard influenced their personal planning and behaviour. High Likert score ratings were given for clarity, ease of use and overall end-user experience, with no differences seen across the various age groups surveyed.

**Conclusion:**

The study demonstrated that both the availability of data and an understanding of end-user need is critical when developing and delivering public health tools that may ultimately garner public trust and influence individual behaviour.

**Supplementary Information:**

The online version contains supplementary material available at 10.1186/s12889-022-14657-w.

## Background

Public health dashboards have been used in the past to communicate, manage and guide local responses to outbreaks, epidemics, and a variety of health conditions [1]. The concept of a ‘visualization dashboard’ is ubiquitous, though, and is found across almost every industry [2]. It is formally defined as “a predominantly visual information display that people use to rapidly monitor current conditions that require a timely response to fulfill a specific role”. This definition, therefore, spans the full spectrum by which information is shared from single page static reports to the more recent evolution of dynamic, interactive, and engaging digital dashboards.

Over the last decade, public health officials have increasingly employed dashboards to guide their response to outbreaks and epidemics [1]. Several examples of this utility include the Somalia Polio outbreak of 2013–2014, the Ebola epidemic of Liberia, Sierra Leone, and Guinea in 2014–2016, and surveillance of the HIV epidemic in New York [3–5]. These examples illustrated the value of leveraging intelligence to rapidly respond to worsening adverse conditions, particularly in the context of an epidemic, through constant and reliable local monitoring [1].

More recently, the ongoing COVID-19 pandemic provided fertile soil within which dashboards proliferated. Judson et al*.* (2022) reported on the variability in COVID-19 reporting systems in Africa and noted that most African countries (98.1%) had some form of official national COVID-19 reporting system in place. Data was communicated in the form of situation reports, press releases, and official dashboards through websites and social media [6].

Beyond access and availability, several features emerged that demonstrated if data available, particularly via dashboards, were actionable. These features included providing data closer to the end-user’s locale, breaking down the population into subgroups, linking time trends to policy decisions, managing the type and volume of data being shown, knowing the audience and their information needs and using visual cues and storytelling as appropriate [7]. Both the availability and quality of public dashboards, therefore, plays an important role in achieving impact on individual decision-making and behaviour change, particularly as it pertains to an African setting.

In the Western Cape, South Africa, the province’s Premier officially launched a COVID-19 dashboard on 30 April 2020 in an online digital conference [8]. The launch highlighted that the dashboard would be a means to ensure transparency and accountability throughout the pandemic [9]. Furthermore, the Premier stated that the dashboard will provide data at a subdistrict level with the belief that this would ensure citizens can protect themselves and their families based on the most recent and correct COVID-19 information at hand [10]. The web-based desktop dashboard was designed, developed, and launched seven weeks after the first provincial case was identified and at a time when the province had recorded a total of only 2,371 COVID-19 cases. The aim of this study was to evaluate the quality, access and end-user experience of the public COVID-19 dashboard that emerged in the Western Cape province, South Africa [11].

## Methods

### Study design and population

The study was conducted by health officials from the provincial government of the Western Cape. The data used in this evaluation study came from two sources. Firstly, we obtained retrospective aggregate data on viewership over time for the first year since launch of the COVID-19 dashboard (30 April 2020 – 29 April 2021) from background analytics running on the dashboard. This included number of daily views and country of origin of viewer which was further aggregated and mapped to respective continental regions. We compared viewership over time by comparing the frequency of views by provincial COVID-19 wave period including the initial two weeks of launch (30 April – 14 May 2020), wave 1 (15 May – 31 August 2020), trough 1 (1 September – 15 October 2020), wave 2 (16 October 2020 – 28 February 2021), and trough 2 (1 March – 29 April 2021). Secondly, we conducted a cross-sectional survey where the target audience was adult users of the public Western Cape COVID-19 dashboard at one year post the initial launch. This primary data was collected via an online digital survey tool accessible on the dashboard through an opt-in manner of delivery and was available for a two-week period between 6 – 21 May 2021. Recruitment was performed through purposive sampling with an invitation to participate as an on-screen pop-up when users engaged with the dashboard. The minimal sample size was calculated using the Kish Leslie formula [12] and was found to be 246 participants for the cross-sectional study based on an assumed proportion accessing the dashboard once a week or more at ~ 20% of all regular viewers. This combined approach of web traffic analysis and an online survey was based on Albert and Tullis’ (2013) work in measuring the user experience [13]. Both approaches provide quantitative insight into participant behaviour and attitudes in a relatively short space of time and is ideal for participants who are geographically dispersed as was the case for the present study’s target sample.

### Survey tool

The self-administered, anonymous questionnaire (see Supplementary File 1) included a total of 13 questions. Demographic variables included age, gender, occupational status, occupation, and location. Access and frequency of use were operationalized through categorical variables in terms of devices used to access the COVID-19 dashboard and how often it was accessed. Quality and impact of the dashboard were operationalized by several variables including: binary variable for impact on personal planning and behaviour, categorical variables for personal purpose and dashboard element most frequently used, five-point Likert scale questions on ease of use, and clarity and understanding, and a ten-point Likert scale for overall rating of end-user experience. Differing Likert scales were used with odd number scoring, allowing for a neutral midpoint score, and even number scoring, ensuring a choice be made in terms of a positive or negative end-user experience [13]. Importantly, the question on dashboard element most frequently used was supplemented with images of the relevant dashboard element to ensure understanding of what was referred to. All questions were mandatory to ensure data completeness.

### Data processing and statistical analysis

Data were analysed using RStudio with data visualizations supplemented by DATAtab, Datawrapper and Power BI. We conducted descriptive and bivariate analyses to describe the population sample. We performed descriptive analyses for frequency of viewers per continental region as well as survey participant characteristics. We applied one-way ANOVA and Kruskal Wallis tests to the normal and non-normal distributed variable categories of COVID-19 wave period for viewership over time respectively and Mann–Whitney U test was applied to the non-normal distributed variables of viewership by weekday vs weekend variability. We used the chi-square test or Fisher’s exact test to assess the associations between reported device most often used and occupational status, reported frequency of use and age category, and reported impact on personal planning and dashboard element most often used. We calculated one-way ANOVA for the mean differences between age groups and Likert scale responses. Importantly, age was aggregated to four categories for the purposes of bivariate analysis. The STROBE (Strengthening the reporting of observational studies in epidemiology) standard was used to ensure that all relevant reporting was reflected [14].

### Ethical considerations

The study was approved by the University of Cape Town Human Research Ethics Committee (HREC REF 260/2021). Informed consent was obtained from all respondents by signed digital agreement prior to opting into performing the self-administered survey questionnaire.

## Results

### Viewership metrics

**Fig. 1:**
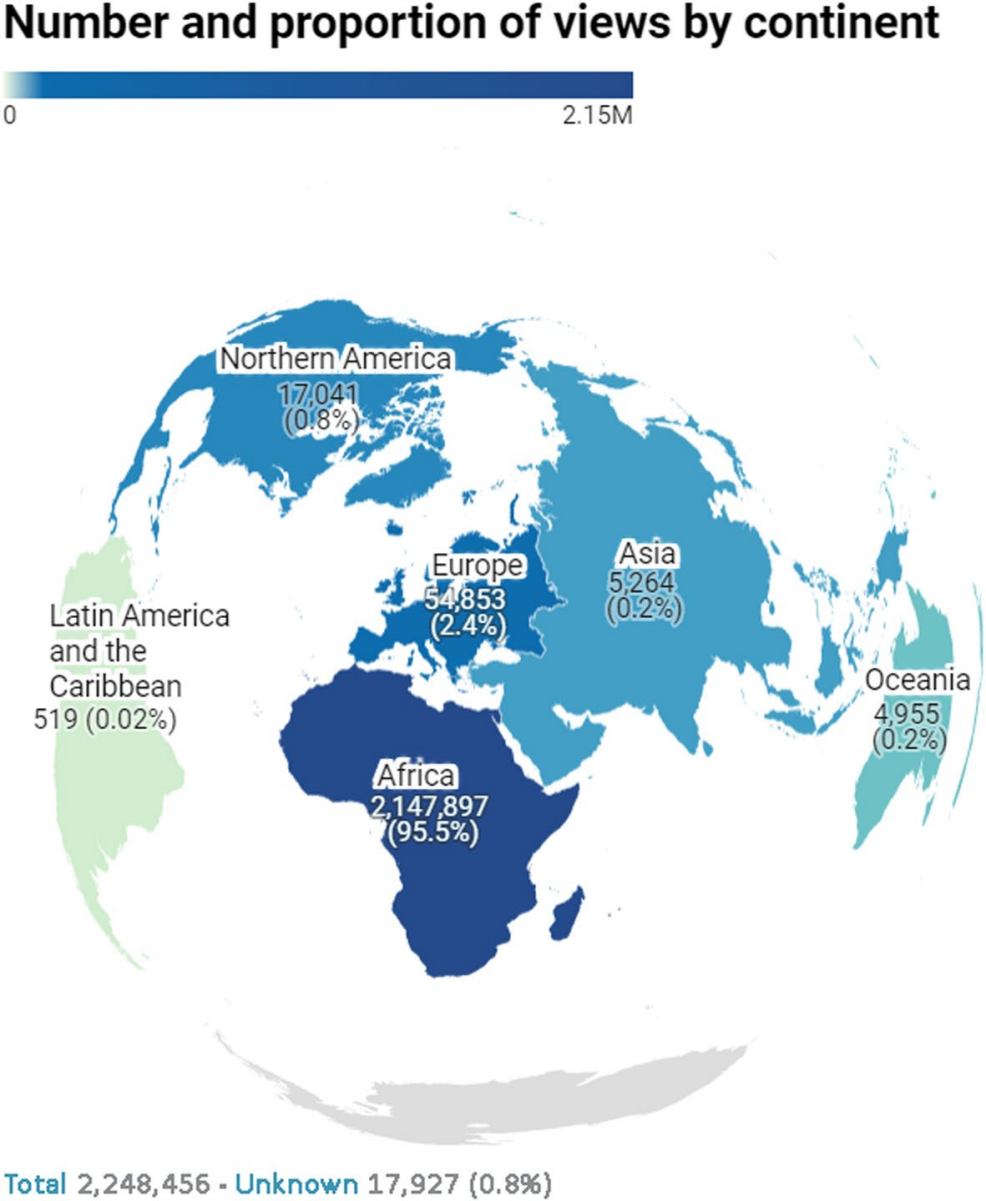
COVID-19 public dashboard views by continental region

**Fig. 2:**
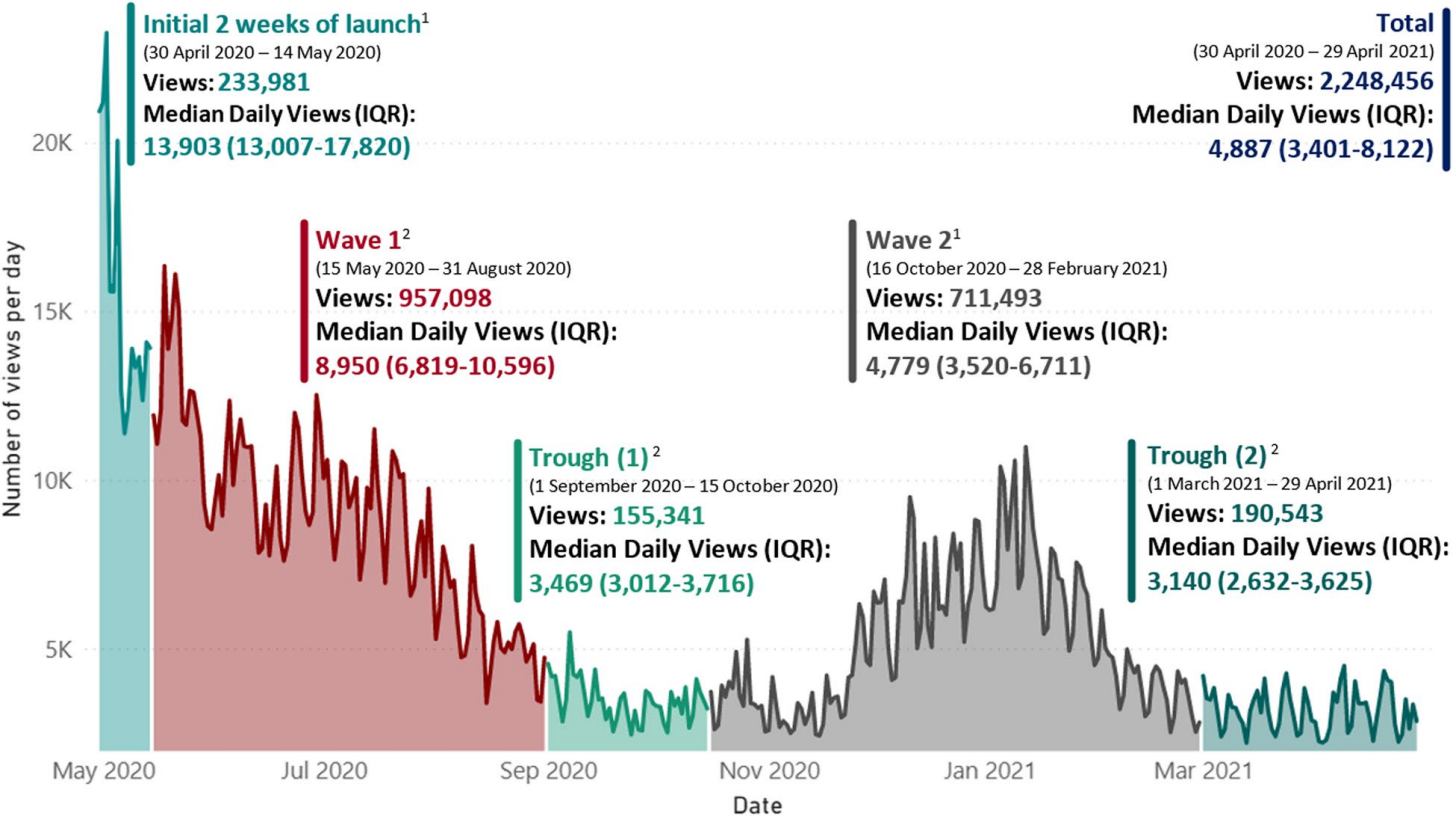
Number of views per day by COVID-19 wave periods and date. ^1^The Shapiro–Wilk test confirmed a non-normal distribution by noting a *p*-value 0.014 for Initial 2 weeks of launch and < 0.001 for Wave 2. A Kruskal–Wallis test for these non-normally distributed periods showed that there is a significant difference between these categorical variable COVID-19 Wave Periods in relation to the variable Number of Views *p* =  < 0.001. ^2^The Shapiro–Wilk test confirmed a normal distribution with *p*-values of 0.129 for Wave 1, 0.105 for Trough (1), and 0.073 for Trough (2). A one-factor analysis of variance (ANOVA) for these normally distributed periods has shown that there is a significant difference between these categorical variable COVID-19 Wave Periods and the variable Number of views per day F = 164.87, *p* =  < 0.001

During the first year (30 April 2020 – 29 April 2021) after the launch the provincial COVID-19 public dashboard had attracted a total of 2,248,456 views. The breakdown by continental region (Fig. 1) shows that 95.5% of these views have come from Africa, followed by Europe and Northern America with a total of 54,853 and 17,041 total views respectively. These were total normal views and could not discern unique individuated viewership. The pattern of daily viewership differed across the various wave periods (Fig. 2). The first 2 weeks post launch saw the highest median number of daily views at 13,903 (IQR: 13,007 – 17,820). Interest waned over the Wave 1 period with a median daily viewership of 8,950 (IQR: 6,819 – 10,596) until a plateau in the trough (1) period. Both trough (1) and (2) periods saw similar median daily views of 3,469 and 3140 respectively (*p* = 0.516). Median daily views in Wave 2 were 4,779 (IQR: 3,520—6,711) with a maximum at 10,984 coinciding with the peak of COVID-19 cases in Jan 2021. The distribution of viewership by day of week over the first year showed higher interest during weekdays with the lowest median daily views on Saturdays and Sundays. The weekday group had higher values for number of views (Median = 5,142) than the weekend group (Median = 4,104; *p* < 0.001).

### Sociodemographic characteristics

A total of 794 individuals responded to the survey invitation during the two-week period of recruitment at one year post the initial launch. Table 1 shows the sociodemographic characteristics of the participants (*n* = 794). Nearly two thirds (63.3%) of respondents were between 40–69 years old with similar proportions seen across both male and female groups [60–69 years old (22.4%), 40–49 years old (21.3%), and 50–59 years old (19.6%)]. Most participants (63.0%) were employed followed by retirees (28.3%). Far fewer participants reported being unemployed or students (5.0% and 3.7% respectively). Most respondents reported their country of origin as South Africa (96.1%) with smaller numbers from the other countries [United States (0.9%), Germany (0.4%) and the United Kingdom (0.3%)]. Reported devices most often used to access the dashboard included a desktop or laptop (65.5%) followed by mobile phones (26.7%) and tablets (7.8%).

**Table 1:** Survey participant characteristics (*n* = 794)

**Participant Characteristics**^a^	**Gender**			
	**Male** **(*n* = 453)**	**Female** **(*n* = 324)**	**Prefer not to say** **(*n* = 17)**	**Total** **(*n* = 794)**
**Age Group**				
18 to 29 years old	24 (3.0%)	18 (2.3%)	2 (0.3%)	44 (5.5%)
30 to 39 years old	70 (8.8%)	66 (8.3%)	5 (0.6%)	141 (17.8%)
40 to 49 years old	92 (11.6%)	72 (9.1%)	5 (0.6%)	169 (21.3%)
50 to 59 years old	88 (11.1%)	64 (8.1%)	4 (0.5%)	156 (19.6%)
60 to 69 years old	108 (13.6%)	69 (8.7%)	1 (0.1%)	178 (22.4%)
70 to 79 years old	61 (7.7%)	30 (3.8%)	0 (0.0%)	91 (11.5%)
> 80 years old	10 (1.3%)	5 (0.6%)	0 (0.0%)	15 (1.9%)
**Occupational Status**				
Employed	286 (36.0%)	200 (25.2%)	14 (1.8%)	500 (63.0%)
Retired	140 (17.6%)	85 (10.7%)	0 (0.0%)	225 (28.3%)
Student	13 (1.6%)	16 (2.0%)	0 (0.0%)	29 (3.7%)
Unemployed	14 (1.8%)	23 (2.9%)	3 (0.4%)	40 (5.0%)
**Device most often used**				
Desktop Computer/Laptop	334 (42.1%)	176 (22.2%)	10 (1.3%)	520 (65.5%)
Mobile Phone	92 (11.6%)	116 (14.6%)	4 (0.5%)	212 (26.7%)
Tablet	27 (3.4%)	32 (4.0%)	3 (0.4%)	62 (7.8%)
**Country of Origin**				
South Africa	430 (54.2%)	319 (40.2%)	14 (1.8%)	763 (96.1%)
Other	23 (2.9%)	5 (0.6%)	3 (0.4%)	31 (3.9%)

^a^Reported as n (%) unless otherwise specified

### Bivariate analysis of survey results

#### Reported devices used by occupational status

**Fig. 3:**
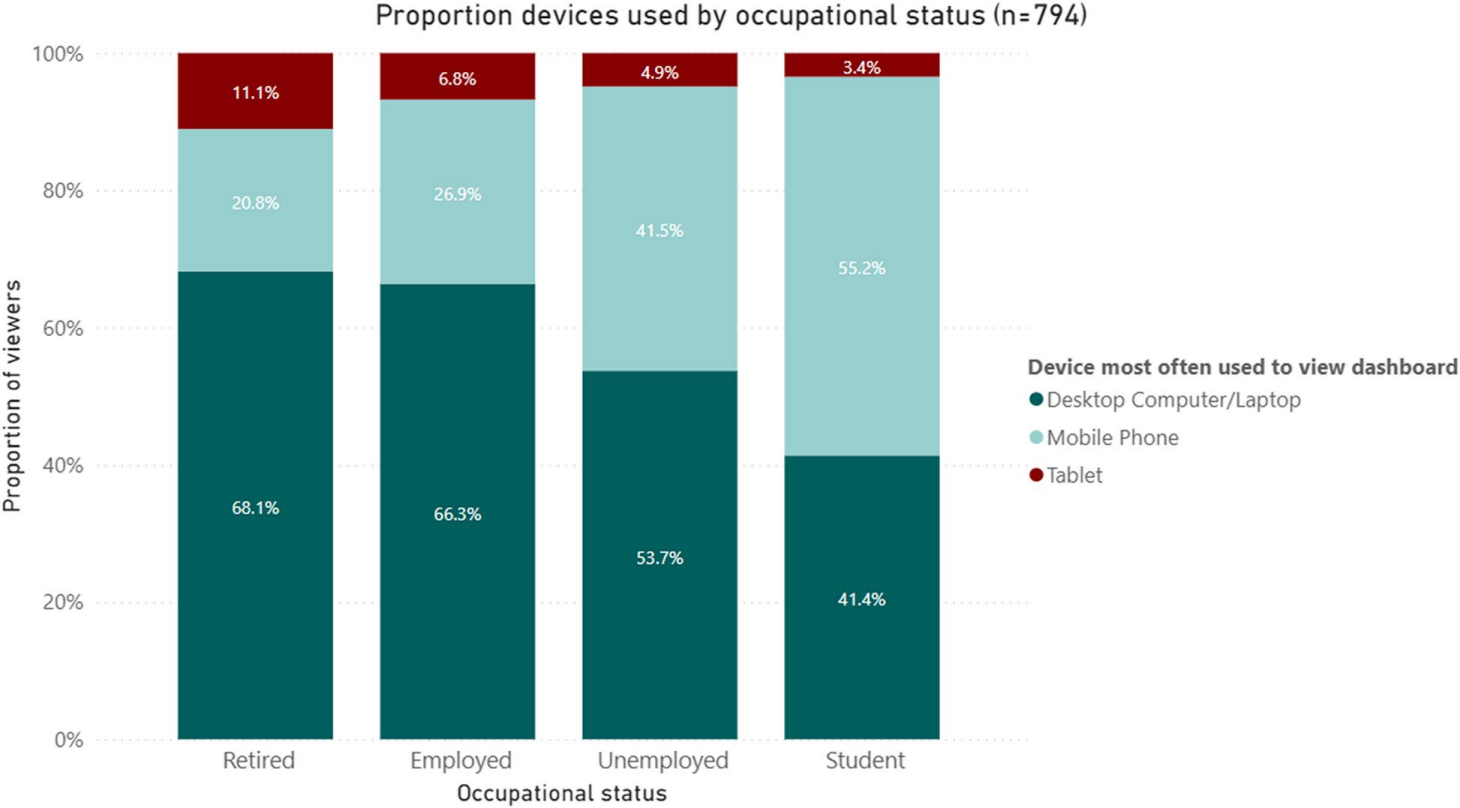
Proportion devices used by occupational status* (*n* = 794). ^*^p-value from Fisher’s exact test 0.001

When comparing the association between devices used to access the dashboard and the occupational status of the participants (Fig. 3), we see that students had the highest tendency to use mobile devices compared to other occupational categories (55.2%). Conversely, 68.1% of all retirees, the highest proportion across all occupational groups, used desktop computers/laptops instead. Unemployed participants were somewhat similar to the student group in terms of access via mobile device usage (41.5%) although they still accessed the dashboard the most through a desktop computer/laptop (53.7%).

#### Reported frequency of use by age category

**Fig. 4:**
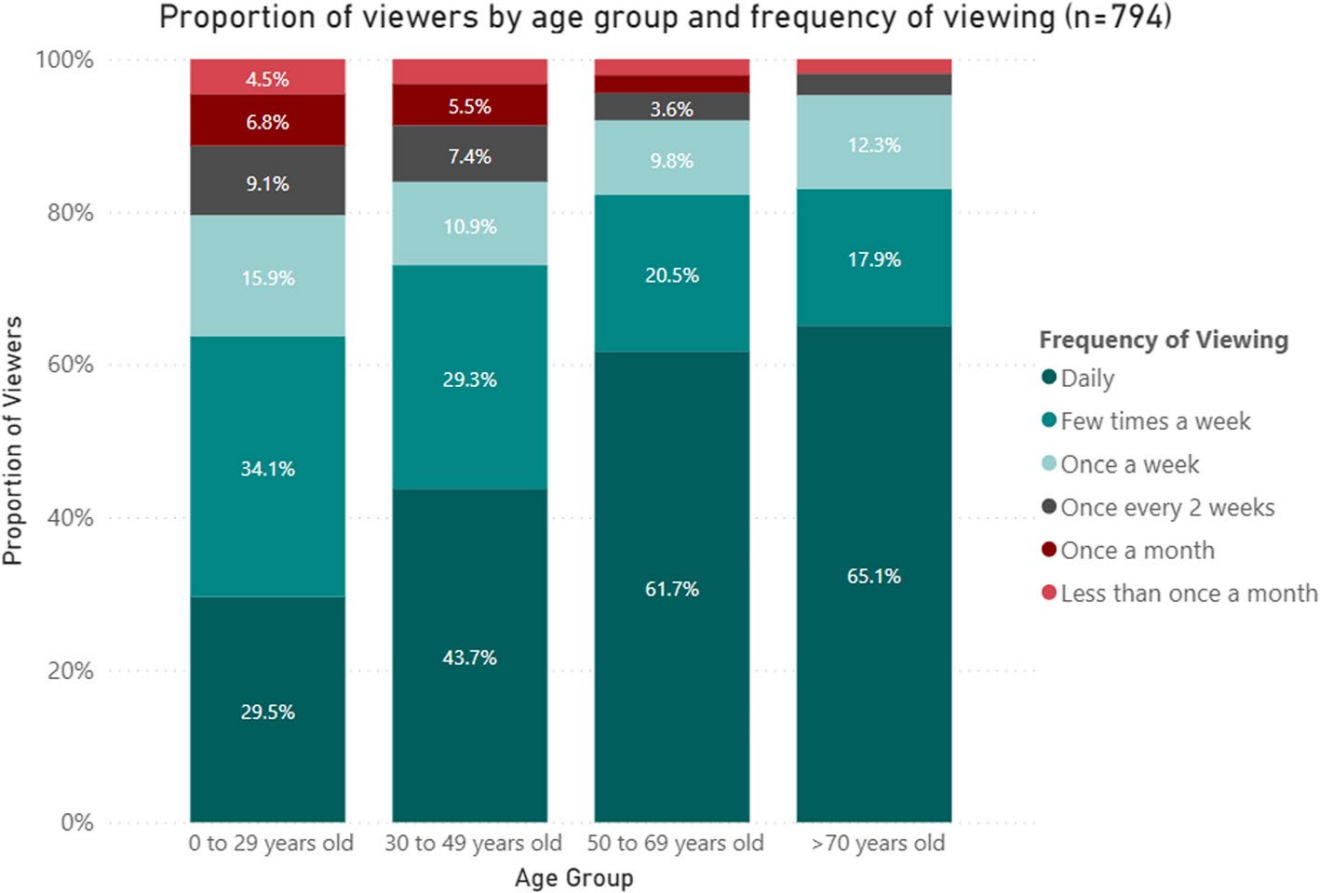
Proportion of viewers by age group and frequency of viewing* (*n* = 794). ^*^p-value from Fisher’s exact test < 0.001

We found that participants in the older age categories viewed the dashboard more frequently (Fig. 4). Of those aged > 70 years old, 65.1% reported viewing the dashboard daily, with a further 17.9% viewing it a few times a week. Frequency of viewing decreased with age with daily viewership found to be 61.7% in 50 to 69 year olds, 43.7% in 30 to 49 year olds and 29.5% in < 29 year olds (*p* < 0.001).

#### Reported impact on personal planning and behaviour by element most often used

**Fig. 5:**
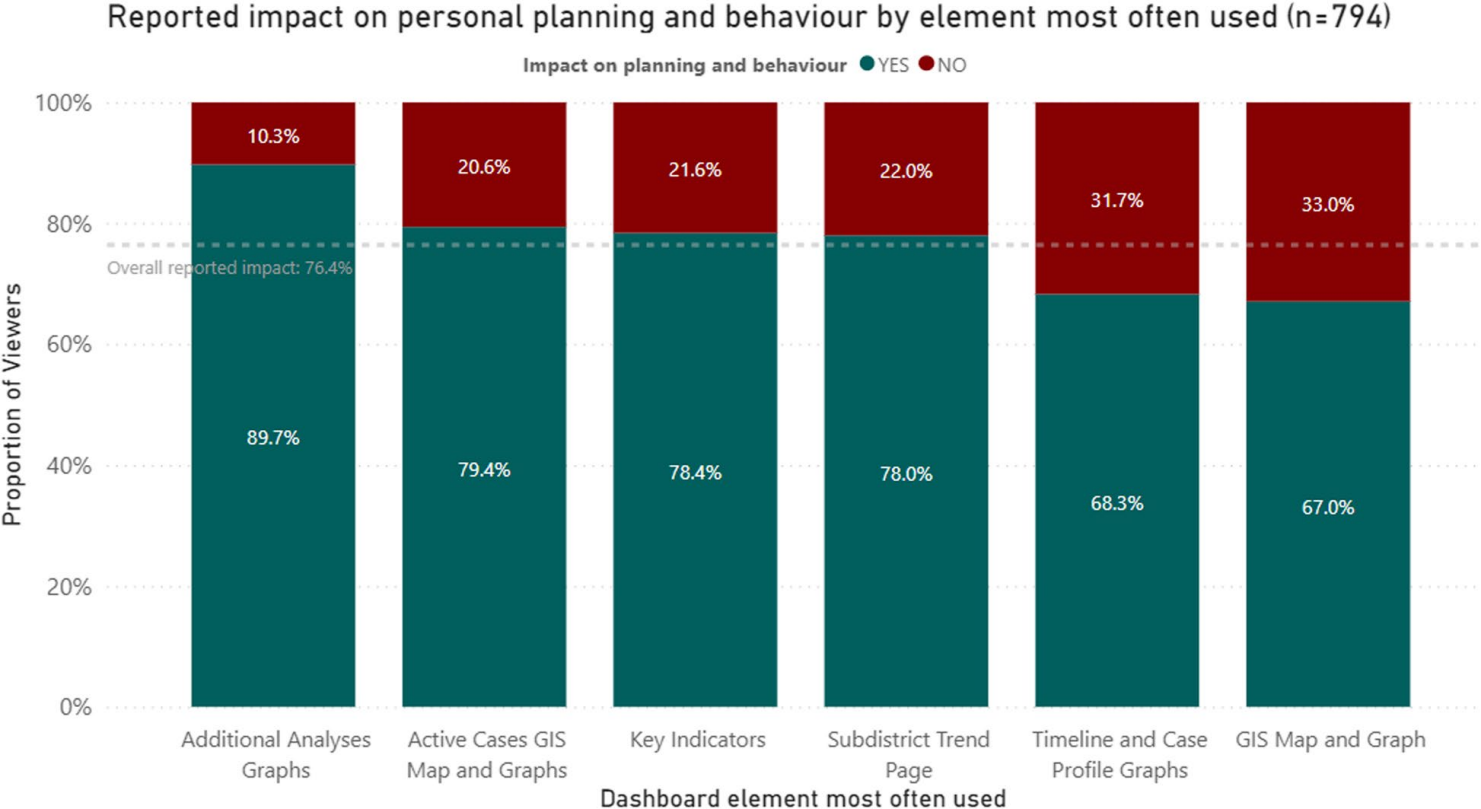
Reported impact on planning and behaviour by element most often used* (*n* = 794). ^*^p-value from Chi.^2^ test 0.006

Overall, 76.4% of all respondents reported that the dashboard influenced their personal planning and behaviour (Fig. 5). Respondents who used the additional analyses graphs most often were more likely to report that the dashboard impacted on their planning and behaviour (89.7%) compared to those who used the GIS map and graph (67.0%; *p* = 0.006).

#### Clarity and understanding, ease of use and overall rating scores by age group

Median Likert-scale ratings of end-user experience were 4/5 for clarity and understanding, 4/5 for ease of use and 8/10 for overall rating with no difference by age category (Table 2).

**Table 2:** Clarity and understanding, ease of use and overall rating scores by age group (*n* = 794)

		**Age Group**					
		**0 to 29** years old	**30 to 49** years old	**50 to 69** years old	** > 70** years old	**Total**	*p*-value*
	Frequency	44	310	334	106	794	
**Clarity and understanding (1–5)**	Mean	4.02	4.02	3.94	4	3.98	0.778
	Median	4	4	4	4	4	
**Ease of use (1–5)**	Mean	4.02	4.04	3.97	3.87	3.98	0.506
	Median	4	4	4	4	4	
**Overall rating (1–10)**	Mean	7.98	7.96	7.92	7.68	7.91	0.627
	Median	9	8	8	8	8	

A one-factor analysis of variance has shown that there is no significant influence between the categorical variable Age Group and the variables Clarity and understanding (1–5) F = 0.37, *p* = 0.778, Ease of use (1–5) F = 0.78, *p* = 0.506, and Overall rating (1–10) F = 0.58, *p* = 0.627

^*^p-values calculated with one-way ANOVA test

## Discussion

The Western Cape public COVID-19 dashboard was well-used in the first year of the pandemic with 2,248,456 views, predominantly by South Africans, with positive end-user experiences.

Firstly, it is important to acknowledge that the delivery of the dashboard was well-communicated to the public by senior political figures at the outset and only 7 weeks after the first case was identified in the Western Cape, South Africa. There was significant media coverage with a resultant highest median daily viewership in the first two weeks of launch. Secondly, most dashboard viewers were from Africa/South Africa. This was reflected in both the aggregate viewership over the first year and in the profile of respondents from the cross-sectional survey. This is reassuring as it reaffirms the statement from Ivanković et al. [7] that a feature of a highly actionable dashboard is one that provides data close to the end-user’s locale. Interestingly, the viewership from North America and Europe are also similarly represented in proportion in both viewership data and sample population from the respondent feedback.

Furthermore, the pattern of viewership over the wave 1, 2 and trough periods provide insight into the differentiated need based on public concern at the time. The increase in daily viewership over the wave 2 period, in particular, provided an indication that the dashboard was referred to as a trustworthy source of information with increased utility during a COVID-19 wave as opposed to between it. The pattern of access also showed an interesting weekday preponderance with lower access over weekends. This may relate to access to the relevant tools needed to view the dashboard as most respondents reported using a computer/laptop to view it or potentially to differences in weekday vs weekend behaviour patterns as it relates the utility of analytic type tools.

The association between devices used most often and occupational status revealed an interesting phenomenon and reflected the characteristics within the underlying population groups. Students accessed the dashboard via their mobile devices far more than any other occupational group. This may be an indication of both comfort in using a mobile device for the purposes of health information and epidemic surveillance as well as access to a mobile device more so than a computer/laptop [15]. A similar picture of increased mobile device access was seen for unemployed participants, but this may be an indication of device access more than comfort in use. Retirees and employed participants displayed a differing affinity to access with the majority opting for a desktop computer/laptop.

The association between frequency of viewing and age groups provided insight into the interest level from the various population groups and potentially toward their motivation for access. There was a consistent pattern of increasing frequency of viewership per increasing age band with the highest frequency of access by those aged > 70 years old. This is, in fact, contrary to research that reported a digital divide between younger groups having higher access and frequency of use to COVID-19 digital tools compared to elderly populations [16]. During the early months of the COVID-19 pandemic there was growing evidence supporting the hypothesis of increased disease severity with increased age [17]. This was confirmed and communicated with local evidence supporting the hypothesis [18]. The increased frequency of viewership in the older age bands in this study may, therefore, be mimicking either the underlying population concern from those most at risk of severe disease or the relative available time in this occupational group to access digital tools.

The fact that just over three quarters of respondents reported that the dashboard had an impact on their personal planning and behaviour speaks to the utility of tools like this amid a pandemic. When overlaying the association between those that reported impact and the element they most often used, it provided an indication to where the most value potentially lies. The results of this study showed that the additional analyses page conferred the highest association with a positive impact on personal planning and behaviour. When looking at what this area contains on the dashboard it becomes clear that it fulfils many of the criteria for a highly actionable dashboard as described by Ivanković et al. [7] including managing the type and volume of data being shown as well as providing sufficient visual cues along with graphs to better explain what is being seen. The engagement necessary to reach this part of the dashboard also seems to indicate that public users are comfortable navigating interactivity in design.

In terms of quality metrics, the dashboard was given relatively high scores in the areas of clarity and understanding, ease of use and overall rating of end-user experience. The study attempted to see if this experience of quality differed by age group but found no such difference with both elderly and young users reporting a similar positive experience.

This evaluation provides insight into the necessity for development of public health data tools within an environment of a health priority as well as the end-user characteristics and needs as it pertains to access, understanding and quality of dashboard tools to communicate the relevant health data. This understanding has implications for public health officials and is pertinent as we transition out from a COVID-19 era and look to apply some of the lessons learnt during COVID-19 towards other communicable and non-communicable diseases.

This study has several limitations. Firstly, although we did not specifically enquire about the end-users’ socio-economic status, we did ask their employment status and the proportions did not seem to align with the demographic characteristics of the Western Cape. Considering that the adult population in the province is ~ 4.8 million with an unemployment rate of 25.8%, the viewership was potentially skewed to higher income, employed cohorts, and low relative to the full adult population [19]. This speaks to the role of dashboards and their relative importance in a context where many people may not have the capacity to access it or plan their personal behaviour in relation to it. This does not negate the importance and value of the dashboard, but it is important to acknowledge the context which is markedly different to a high-income country where one may expect most of the population to access and use tools such as this. Secondly, due to the nature of online recruitment the sampling approach could not avoid the potential bias of subjective selection or nonresponse bias. In other words, there may have been a difference in those that responded to those that did not. Secondly, the timing of the cross-sectional survey was done at one year post the initial launch. This may confer selection bias both in terms of only targeting those that are still making use of the dashboard one year later and that find value in it as well as those that tend to use it more frequently than others. Thirdly, there may have been a combined selection and information bias since the survey was done during an trough period and the type of recall and type of participant may have been different should it have been asked during a wave. Last, the dashboard was not designed for easy mobile device use and may have biased the responses in terms of type of device most often used. A change in the design towards a more mobile-friendly interface may have shifted the responses towards a different modality of access.

## Conclusion

The need for research into the delivery of intuitive, easy-to-understand and timely health data for public use is rapidly growing. This is an important consideration as we emerge toward a post-COVID-19 era and need to leverage the lessons for an African context that is often sparse in terms of data availability. The present study findings, though, demonstrate that it is both availability of data and an understanding of end-user need that is important to develop and deliver quality and appropriate health data tools that may ultimately garner public trust, ensure accountability, and influence individual behaviour.

## Supplementary Information

**Additional file 1: Supplementary Table 1.** Questionnaire with possible response options. **Supplementary Table 2.** Number of views by continental region. **Supplementary Table 3.** Number of views per day by COVID-19 period (30 April 2020 – 29 April 2021). **Supplementary Figure 1.** Number of views per day by COVID-19 wave period. **Supplementary Figure 2.** Median number of views by day of week. **Supplementary Figure 3.** Number of views per day by weekday vs weekend*. **Supplementary Table 4.** Reported devices used by occupational status* (n=794). **Supplementary Table 5.** Reported frequency of use by age category* (n=794). **Supplementary Table 6.** Reported impact on personal planning and behaviour by element most often used* (n=794). **Supplementary Table 7.** Reported purpose of dashboard for participants* (n=1279).

## Data Availability

The datasets used and analysed during the current study are available from the corresponding author on reasonable request.

## Abbreviations

COVID-19: Coronavirus Disease
GIS: Geographic Information System
HIV: Human Immunodeficiency Virus
IQR: Interquartile Range
