# Supplementary material for: Evaluation of a public COVID-19 dashboard in the Western Cape, South Africa: a tool for communication, trust, and transparency
Source: BMC Public Health. 2022 Dec 29;22:2453. doi: 10.1186/s12889-022-14657-w (PMC9797888; doi:10.1186/s12889-022-14657-w)
Supplement: Supplementary file 1 — Additional file 1: Supplementary Table 1. Questionnaire with possible response options. Supplementary Table 2. Number of views by continental region. Supplementary Table 3. Number of views per day by COVID-19 period (30 April 2020 – 29 April 2021). Supplementary Figure 1. Number of views per day by COVID-19 wave period. Supplementary Figure 2. Median number of views by day of week. Supplementary Figure 3. Number of views per day by weekday vs weekend*. Supplementary Table 4. Reported devices used by occupational status* (n=794). Supplementary Table 5. Reported frequency of use by age category* (n=794). Supplementary Table 6. Reported impact on personal planning and behaviour by element most often used* (n=794). Supplementary Table 7. Reported purpose of dashboard for participants* (n=1279). [file 12889_2022_14657_MOESM1_ESM.pdf]

Supplementary File

Study Title: Evaluation of a Public COVID-19 Dashboard in the Western Cape, South Africa: a tool for communication, trust, and transparency

Supplementary Table 1: Questionnaire with possible response options

Question	Variable Type	Possible response options	Comments
What device do you view the dashboard on?	Categorical: Nominal	• Mobile Phone<br>• Desktop Computer/ Laptop<br>• Tablet	
How often do you check the dashboard?	Categorical: Ordinal	• Daily<br>• Few times a week<br>• Once a week<br>• Once every 2 weeks<br>• Once a month<br>• Less than once a month	
How easy was the Western Cape COVID-19 dashboard to use?	Likert Scale (Numeric: Discrete)	• 1 to 5	
How clear and understandable was the information on the Western Cape COVID-19 dashboard?	Likert Scale (Numeric: Discrete)	• 1 to 5	
Has the dashboard influenced your personal planning or behaviour in the past?	Binary	• Yes<br>• No	Note: The question is non-mandatory and should the participant prefer not to answer they can move to the following question.
What purpose does the dashboard serve for you?	Categorical: Nominal	• Getting an update of Key Indicators<br>• Viewing historical data<br>• Ad hoc analysis<br>• Reporting for the media<br>• Reporting for a particular industry<br>• Other	Note: Other allows for a free text option as well.
What is your current occupational status?	Categorical: Nominal	• Employed<br>• Student<br>• Unemployed<br>• Retired	

What is your occupation?	Categorical: Nominal	• Look-up table to OFO (Organizing Framework for Occupations 2019) as reference to all occupations	Note: This only gets asked if the participant states that they are employed in the previous question
Which dashboard element do you use the most?	Categorical: Nominal	• Key Indictors<br>• Timeline and Case Profile Graphs<br>• GIS Map and Graph<br>• Subdistrict Trend Page<br>• Additional Analyses Graphs<br>• Active Cases GIS Map and Graphs	Note: These options will be provided with images representing the various options.
Overall, on a scale from 1 to 10, how would you rate the Western Cape COVID-19 Dashboard?	Likert Scale (Numeric: Discrete)	• 1 to 10	
Which gender do you identify as?	Categorical: Nominal	• Male<br>• Female<br>• Prefer not to say	
What age group do you fall in?	Categorical: Ordinal	• 0 to 17 years old<br>• 18 to 29 years old<br>• 30 to 39 years old<br>• 40 to 49 years old<br>• 50 to 59 years old<br>• 60 to 69 years old<br>• 70 to 79 years old<br>• >80 years old	
Which city do you come from?	Categorical: Nominal	• All cities in the world	Note: This field will be include a lookup for all cities in the world
If you'd like to receive a copy of the results and analysis from this survey, please provide your email address. If not, simply click submit.	Email Address	• Free text with email validation	Note: This is not for analysis purposes, will be immediately separated from the rest of the dataset and will only be used to communicate findings from the survey back to the participant.

Supplementary Analyses

Supplementary Table 2: Number of views by continental region

		COVID-19 dashboard views	
		**Number of views**	**Frequency**
**Continental region**	Africa	2,147,897	95.53%
	Europe	54,853	2.44%
	Northern America	17,041	0.76%
	Asia	5,264	0.23%
	Oceania	4,955	0.22%
	Latin America and the Caribbean	519	0.02%
	Unknown	17,927	0.80%
	**Total:**	**2,248,456**	**100%**

Supplementary Table 3: Number of views per day by COVID-19 period (30 April 2020 – 29 April 2021)

		COVID-19 Wave Period				
		**Initial 2 weeks of launch[3]**	**Wave 1[2]**	**Interwave (1)[2]**	**Wave 2[3]**	**Interwave (2)[2]**
	n	15	109	45	136	60
**Number of views per day**	Median[1]	13,903	8,950	3,469	4,778.5	3,140
	Sum	233,981	957,098	155,341	711,493	190,543
	Std. Deviation	3,816.2	2,788.4	630.3	2,089.9	627.8
	Minimum	11,383	3,385	2,445	2,415	2,202
	Maximum	23,255	16,340	5,484	10,984	4,492

[1]p-values calculated with one-way ANOVA test or Kruskal-Wallis test all showed statistical significance $p<0.05$
[2]The Shapiro-Wilk test confirmed a normal distribution with a p-value >0.05 for Wave 1, Interwave (1), Interwave (2). A one-factor analysis of variance (ANOVA) for these normally distributed periods has shown that there is a significant difference between these categorical variable COVID-19 Wave Periods and the variable Number of views per day $F = 164.87$, $p = <.001$
[3]The Shapiro-Wilk test confirmed a non-normal distribution by noting a p-value <0.05 for Initial 2 weeks of launch and Wave 2. A Kruskal-Wallis test for these non-normally distributed periods showed that there is a significant difference between these categorical variable COVID-19 Wave Periods in relation to the variable Number of Views p=<.001.

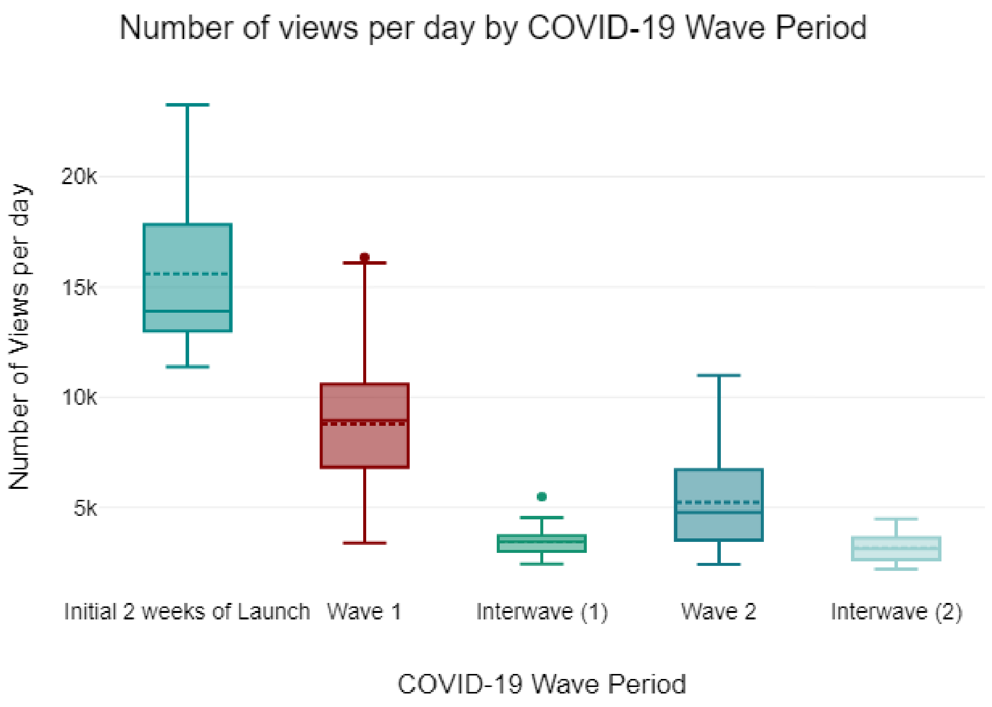

Supplementary Figure 1: Number of views per day by COVID-19 wave period

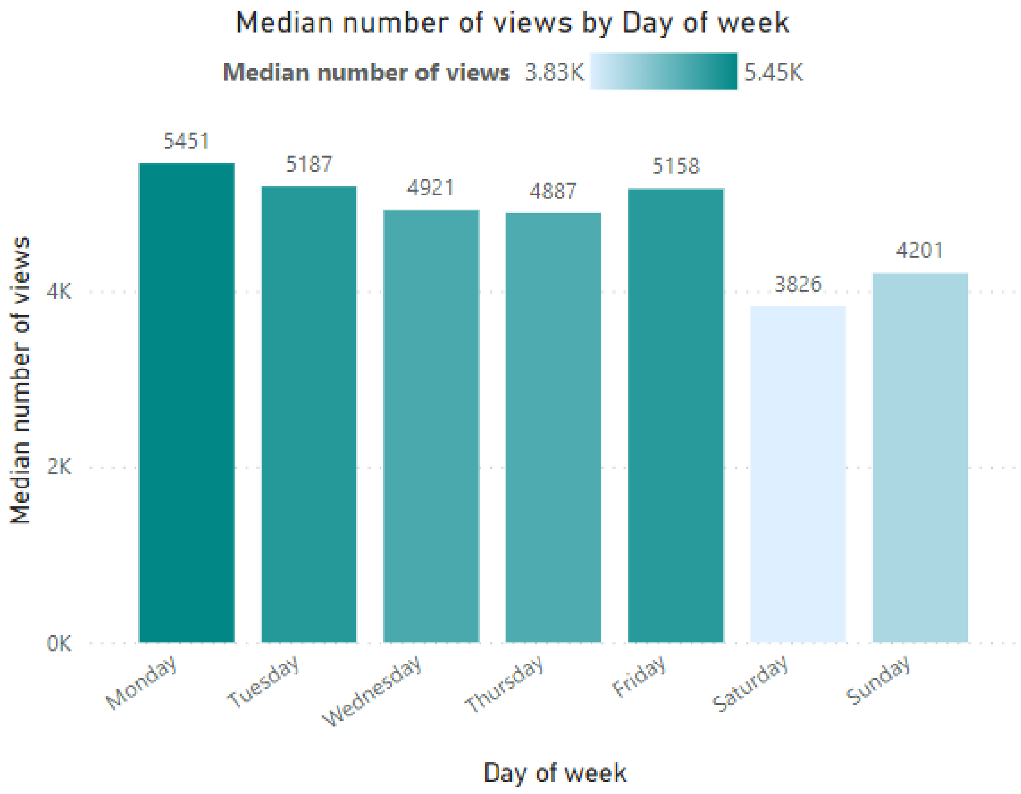

Supplementary Figure 2: Median number of views by day of week

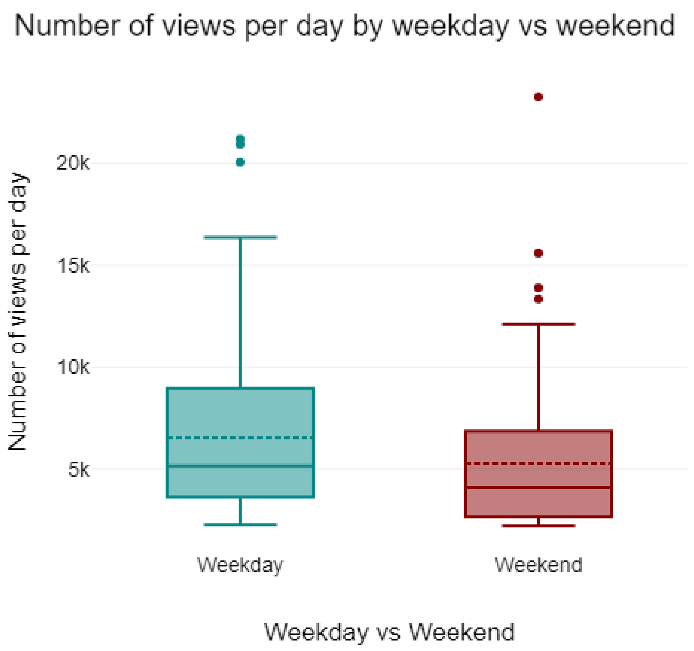

Supplementary Figure 3: Number of views per day by weekday vs weekend*

*The Shapiro-Wilk test confirmed a non-normal distribution by noting a $p<0.05$.
p-value calculated with Mann-Whitney U-Test showed statistical significance between the groups $p<0.05$

Supplementary Table 4: Reported devices used by occupational status* (n=794)

		Occupational Status									
		**Employed**		**Retired**		**Student**		**Unemployed**		**Total**	
		n	%	n	%	n	%	n	%	n	%
**Device Used**	**Desktop Computer / Laptop**	332	41.81%	154	19.4%	12	1.51%	22	2.77%	520	65.49%
	**Mobile Phone**	134	16.88%	46	5.79%	16	2.02%	16	2.02%	212	26.7%
	**Tablet**	34	4.28%	25	3.15%	1	0.13%	2	0.25%	62	7.81%
	**Total**	500	62.97%	225	28.34%	29	3.65%	40	5.04%	794	100%

*p-value from Chi^2 test <0.05

Supplementary Table 5: Reported frequency of use by age category* (n=794)

		Frequency of use												
		Daily		Few times a week		Once a week		Less than once a month		Once a month		Once every 2 weeks		Total
		n	%	n	%	n	%	n	%	n	%	n	%	n
**Age Group**	**0 to 29 years old**	13	29.55%	15	34.09%	7	15.91%	2	4.55%	3	6.82%	4	9.09%	44
	**30 to 49 years old**	136	43.87%	90	29.03%	34	10.97%	10	3.23%	17	5.48%	23	7.42%	310
	**50 to 69 years old**	206	61.68%	68	20.36%	33	9.88%	7	2.1%	8	2.4%	12	3.59%	334
	**>70 years old**	69	65.09%	19	17.92%	13	12.26%	2	1.89%	0	0%	3	2.83%	106
	**Total**	424		192		87		21		28		42		794

*p-value from Chi² test <0.05

Supplementary Table 6: Reported impact on personal planning and behaviour by element most often used* (n=794)

		Dashboard has influenced participant's personal planning or behaviour					
		YES		NO		Total	
		n	%	n	%	n	%
**Dashboard element most often used**	**Key Indicators**	242	78.32%	67	21.68%	309	38.92%
	**GIS Map and Graph**	59	67.05%	29	32.95%	88	11.08%
	**Additional Analyses Graphs**	60	89.55%	7	10.45%	67	8.44%
	**Subdistrict Trend Page**	84	77.78%	24	22.22%	108	13.60%
	**Active Cases GIS Map and Graphs**	76	79.17%	20	20.83%	96	12.09%
	**Timeline and Case Profile Graphs**	86	68.25%	40	31.75%	126	15.87%
	**Total**	607	76.44%	187	23.56%	794	100%

*p-value from Chi² test <0.05

Supplementary Table 7: Reported purpose of dashboard for participants* (n=1279)

What purpose does the dashboard serve for you?	Number of responses	
	Frequency (n)	%
**Getting an update of Key Indicators**	739	57.78%
**Viewing historical data**	277	21.66%
**Ad hoc analysis**	213	16.65%
**Reporting for a particular industry**	36	2.81%
**Reporting for the media**	14	1.09%
**Total**	1279	100%

*Multiple responses were allowed for this question. Free text feedback marked as ‘other’ was removed for this analysis.
